# Cancer risk after preeclampsia: a cohort study in two Nordic populations

**DOI:** 10.1038/s44276-025-00162-1

**Published:** 2025-07-03

**Authors:** Golbarg Vesterlund, Xinhe Mao, Mika Gissler, Manuchehr Abedi-Valugerdi, Tiina Skoog, Seppo Heinonen, Pär Sparen, Karin Pettersson, Juha Kere, Kamila Czene, Satu Wedenoja

**Affiliations:** 1 Department of Medicine Huddinge, Karolinska Institutet, Stockholm, Sweden; 2 Department of Epidemiology and Biostatistics, Karolinska Institutet, Stockholm, Sweden; 3 Department of Data and Analytics, THL Finnish Institute for Health and Welfare, Helsinki, Finland; 4 Department of Molecular Medicine and Surgery, Karolinska Institutet, Stockholm, Sweden; 5 Department of Laboratory Medicine, Karolinska Institutet, Stockholm, Sweden; 6 Department of Obstetrics and Gynecology, University of Helsinki and Helsinki University Hospital, Helsinki, Finland; 7 Department of Clinical Science, Intervention and Technology, Karolinska Institutet, Stockholm, Sweden; 8 Folkhälsan Research Centre, Helsinki, Finland

## Abstract

**Background:**

Limited evidence suggests that preeclampsia (PE) is associated with reduced cancer risk later in life. We aimed to investigate this using large registry-based cohorts. We hypothesised that enhanced immune activation in PE women, suggested by autoimmune-type reactivity, lowers their subsequent cancer risk.

**Methods:**

Utilising Medical Birth Registry data from Sweden and Finland, we identified 123,495 women with PE and 3,223,537 women without. Data were cross-linked to the national Cancer Registries. Incidence rate ratios with 95% CIs were calculated and adjusted for maternal birth year, age at first birth, and parity.

**Results:**

Overall cancer risk was significantly lower in Swedish PE women (IRR 0.91) but not in Finnish. Lower IRRs in PE women were found in both cohorts for breast (IRR 0.90 and 0.91), cervical (IRR 0.79 and 0.55) and lung cancer (IRR 0.72 and 0.63), while endometrial cancer showed increased incidence (IRR 1.28 and 1.46). Foetal sex had no impact on cancer risk. Among Swedish siblings to PE women, a slight reduction in cancer risk, driven by lower lung cancer incidence (IRR 0.86), was noted.

**Conclusion:**

Our data show a link between PE and subsequent cancer risk, suggesting that shared mechanisms may predispose to PE and influence cancer development.

## Background

Preeclampsia (PE), a hypertensive pregnancy disorder, is a major cause for concern during pregnancy due to its potential complications for both the mother and the foetus. While the delivery cures PE, its consequences on maternal and child health are long-term [1]. Numerous studies have demonstrated an increased risk of subsequent cardiovascular disease [2], stroke [3] and kidney disease in the mother [4], and even an increased risk of hypertension [5]and stroke [6] in the child.

Beyond cardiovascular disease, PE might modulate the risk of cancerous tumours later in life. Several studies, with still inconclusive results, have searched for the link between PE and cancer. While the results vary from reduced cancer incidence [7–9] to no association [10, 11], some studies found an increased risk of cancer after PE [12, 13]. Together, these findings suggest that undetermined mechanisms contribute to human pregnancy success, risk of PE, and development of cancer.

Our study was motivated by the proposed but still disputed variation in the cancer risk after PE. Given the role of immunological maladaptation in PE and cancer [14–17], we hypothesised that enhanced maternal immune responses in PE pregnancy [14, 15, 18] might trade-off with stronger immune responses that protect them from cancer later in life. To test our hypothesis, we conducted a population register-based study using large cohorts from Sweden and Finland, with a total of 3,401,724 women among whom 123,495 with PE. We searched for the association between PE at the first delivery and subsequent cancer risk. We also explored whether this association varies by cancer subtype and whether fetal sex plays a role [19–21]. Furthermore, to investigate whether genetic factors may contribute to the association between PE and cancer, we analysed cancer risk among siblings of women with PE in the Swedish cohort.

## Methods

### Study design and data sources

This registry-based cohort study was approved by the regional ethical board (reference numbers 2012/217-32/2 and 2014/1401-32) in Stockholm, Sweden and by the Finnish Institute for Health and Welfare (THL/2295/5.05.00/2019). No informed consents were required. Research was performed according to the approved guidelines and by the Strengthening the Reporting of Observational Studies in Epidemiology (STROBE) guidelines for cohort studies.

### Study population

We used data from Medical Birth Registries in Sweden and Finland, capturing 98–100% of all deliveries in both countries [22, 23]. In Finland, the data were supplemented with information from the Hospital Discharge Registry. Data on dates of delivery, sex of the newborn, gestational age at birth, maternal comorbidities, and parity (number of previous births) were recorded.

**Fig. 1:**
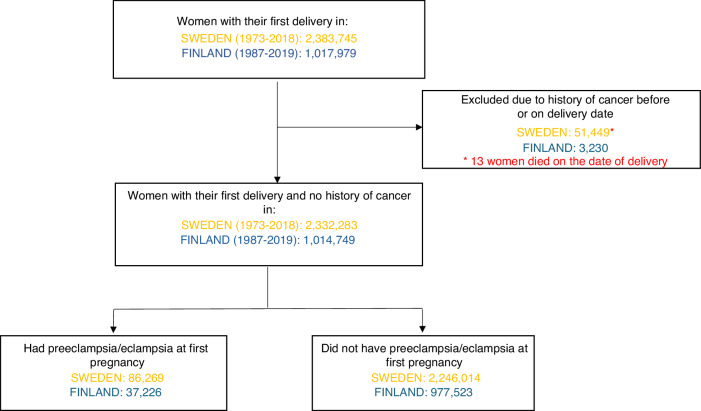
Flowchart of the participants.

We identified 2,383,745 and 1,017,979 index women who gave birth between 1973 and 2018 in Sweden and between 1987 and 2019 in Finland, respectively. The study women were linked based on their personal identification numbers to different national registers, including the Cancer Registry and the Cause of Death Registry in both countries, as well as the Multi-Generation Registry and Migration Registry in Sweden. After linkage, we excluded 51,449 Swedish and 3230 Finnish women who had a cancer diagnosis before their first delivery (Fig. 1). Through linkage with the Multi-Generation Register, we identified 2,046,880 full siblings of Swedish index women.

### Exposure

We defined exposure as the diagnosis of PE at the index woman’s first delivery. Index women with PE were selected using the International Statistical Classification of Diseases and Related Health Problems (ICD) codes, as described by the WHO. For the Swedish data, we used ICD-8 codes (1969–1986): 63703, 63704, 63709, 63710, and 63799; ICD-9 (1987–1996): 642E, 642 F, and 642 G; and ICD-10 (1997–2018): O14 and O15. For the Finnish data, PE cases were selected using ICD-9 (1987–1995): 6424 A, and 6425 A; and ICD-10 (1996-2018): O14.

### Outcomes

Through linkage to the Cancer Registries, data spanning from 1958 to 2018 in Sweden and 1987 to 2019 in Finland, we retrieved data on the dates of first cancer diagnosis and types of cancer in the study women. Through linkage to the Cause of Death Register and the Migration Register (not available for Finland), we were able to censor deaths, and for the Swedish data, migration. In the analyses for index women, they were followed up from the date of the first delivery until the first cancer diagnosis, emigration, death, or December 31, 2018, whichever came first. In the analyses for the index women’s siblings, individuals were followed from birth until first cancer diagnosis, emigration, death, or December 31, 2018, whichever came first.

### Statistical methods

We examined the association between a diagnosis of PE at the first delivery and the risk of subsequent cancer using Poisson regression. Age was used as the timescale in the Poisson regression models. Calendar year, age of women at the first delivery, and parity were used as covariates in the analyses. Maternal birth year was included, as differences in environmental exposures, medical advances, and lifestyle changes across generations might influence cancer risk. Age at the first delivery was also considered, as women who give birth for the first time at an older age may face a higher risk of developing cancers such as breast and ovarian cancer later in life [24, 25]. Additionally, parity was adjusted for, since higher numbers of deliveries have been linked to a reduced risk of certain cancers, particularly breast cancer [26]. In the analyses of the index women’s full siblings, calendar year, age of women at first birth, and the number of sisters who had ever given birth (if applicable) were used as covariates. The number of sisters who had ever given birth was included as a covariate because the probability of observing a sister with a PE birth depends on how many sisters have had the opportunity to give birth. We conducted all analyses using the SAS version 9.4 (SAS Institute Inc.).

## Results

### Descriptive data

The study series involved 2,332,283 Swedish and 1,014,749 Finnish women without cancer at baseline. They were followed up for a median of 21 years in the control group and 18 years in the PE group. During the study period, 294,287 (12.6%) Swedish and 50,090 (4.9%) Finnish women were diagnosed with cancer. The mean age at the cancer diagnosis, 47 years in Sweden and 49 years in Finland, was similar among women with PE and those without (Table 1).

**Table 1:** Characteristics of the study series.

	Sweden		Finland	
	No PE (*n* = 2,246,014)	PE (*n* = 86,269)	No PE (*n* = 977,523)	PE (*n* = 37,226)
**Median follow-up (IQR)**	21.6 (9.3-34.1)	18.7 (8.4-30.2)	21.1 (11.1-30.5)	18.9 (10.6–29.7)
**Mean age at first cancer (SD)**	47.4 ± 13.7	46.6 ± 13.2	49.1 ± 9.4	49.1 ± 9.8
**Median birth year (IQR)**	1966 (1955-1978)	1969 (1959-1979)	1973 (1964–1983)	1973 (1964–1983)
	% (n)	% (n)	% (n)	% (n)
**Age at first birth**				
≤19	5.3 (119,213)	4.8 (4121)	4.4 (43,320)	4.3 (1634)
20–24	27.1 (608,034)	26.0 (22,445)	21.5 (210,309)	21.5 (8007)
25–29	36.2 (813,821)	34.3 (29,627)	35.5 (346,571)	33.4 (12,437)
30–34	22.2 (499,035)	22.9 (19,715)	25.7 (250,822)	24.9 (9264)
35 or more	9.2 (205,910)	12.0 (10,361)	12.9 (126,501)	15.8 (5884)
Unknown	0.0 (1)	0.0 (0)	0.0 (0)	0.0 (0)
**Parity**				
1	20.4 (458,327)	26.6 (22,929)	35.3 (345,433)	39.5 (14,716)
2	48.1 (1,081,112)	47.2 (40,716)	40.7 (398,066)	39.1 (14,571)
3	22.3 (501,558)	19.0 (16,425)	16.7 (163,397)	15.1 (5626)
4	6.3 (141,388)	5.0 (4338)	4.6 (44,824)	4.0 (1478)
5 or more	2.8 (63,629)	2.2 (1861)	2.6 (25,803)	2.2 (835)
**Offspring sex**				
Male	51.4 (1,154,179)	52.1 (44,917)	51.2 (500,356)	51.0 (18,981)
Female	48.6 (1,091,723)	47.9 (41,346)	48.8 (477,122)	49.0 (18,245)
Unknown	0.0 (112)	0.0 (6)	0.0 (45)	0.0 (0)
**Birth weight in first birth**				
<3000	16.7 (376,121)	40.6 (35,037)	15.6 (152,655)	43.6 (16,233)
3000–3999	68.6 (1,540,003)	47.9 (41,301)	69.3 (677,196)	47.5 (17,693)
4000 or more	14.4 (324,086)	11.2 (9621)	15.0 (147,028)	8.8 (3270)
Unknown	0.3 (5804)	0.4 (310)	0.07 (644)	0.08 (30)
**Duration of first pregnancy**				
<37	5.6 (124,707)	22.0 (18,971)	5.3 (51,575)	24.5 (9104)
37 or more	92.6 (2,078,686)	75.9 (65,508)	94.5 (923,267)	75.4 (28,077)
Unknown	1.9 (42,621)	2.1 (1790)	0.3 (2681)	0.1 (45)

PE denotes preeclampsia.

Women with PE had more preterm deliveries (<37 weeks) compared to controls: 22.5% vs. 5.7% in Sweden, and 24.5% vs. 5.3% in Finland. Moreover, newborns of PE women had lower birthweight and the proportion of those weighing less than 3000g was increased: 40.8% vs. 16.8% in Sweden, and 43.6% vs. 15.6% in Finland (Table 1). The higher occurrence of preterm birth and low birthweight in PE pregnancies was expected and largely iatrogenic. Other baseline characteristics including age at the first delivery, parity, and the sex of the offspring, showed no differences in PE and controls in the national cohorts (Table 1).

### Main results

We observed decreased incidence rate ratios for any cancer among women having PE at their first delivery in Sweden (IRR 0.91, 95% CI: 0.90–0.93) but not in Finland (IRR: 0.97, 95% CI:0.92-1.02) (Table 2). Lower incidence rate ratios were observed for several cancer subgroups in both cohorts, including breast cancer (IRR 0.90, 95% CI: 0.86–0.94 in Sweden and IRR 0.91, 95% CI: 0.83–0.98 in Finland), cervical cancer (IRR 0.79, 95% CI: 0.76–0.82 in Sweden and IRR 0.55, 95% CI: 0.19–0.91 in Finland), and lung cancer (IRR 0.72, 95% CI: 0.62–0.84 in Sweden and IRR 0.63, 95% CI: 0.27–0.99 in Finland) (Table 2).

**Table 2:** Association of preeclampsia at the first delivery with subsequent risk of cancer in Sweden and Finland.

		Sweden			Finland		
		No. ppl	No. cases	IRR (95% CI)	No. ppl	No. cases	IRR (95% CI)
**Any cancer**	History of PE						
	No	2,246,014	285,409	1.00 (Ref)	977,523	48,369	1.00 (Ref)
	Yes	86,269	8878	**0.91 (0.90–0.93)**	37,226	1721	0.97 (0.92–1.02)
Cervical cancer^*^	History of PE						
	No	2,246,014	94,686	1.00 (Ref)	977,523	1319	1.00 (Ref)
	Yes	86,269	2762	**0.79 (0.76–0.82)**	37,226	31	**0.55 (0.19–0.91)**
**Breast cancer**	History of PE						
	No	2,246,014	69,005	1.00 (Ref)	977,523	19,598	1.00 (Ref)
	Yes	86,269	2039	**0.90 (0.86–0.94)**	37,226	651	**0.91 (0.83–0.98)**
**Melanoma**	History of PE						
	No	2,246,014	20,217	1.00 (Ref)	977,523	2913	1.00 (Ref)
	Yes	86,269	757	1.07 (0.99-1.15)	37,226	99	0.93 (0.73–1.13)
**Colorectal cancer**	History of PE						
	No	2,246,014	15,250	1.00 (Ref)	977,523	974	1.00 (Ref)
	Yes	86,269	451	0.96 (0.87-1.05)	37,226	31	0.86 (0.50–1.22)
**Ovarian cancer**	History of PE						
	No	2,246,014	8275	1.00 (Ref)	977,523	1214	1.00 (Ref)
	Yes	86,269	278	1.05 (0.93-1.18)	37,226	41	0.92 (0.61–1.23)
**Lung cancer**	History of PE						
	No	2,246,014	8259	1.00 (Ref)	977,523	1165	1.00 (Ref)
	Yes	86,269	171	**0.72 (0.62–0.84)**	37,226	30	**0.63 (0.27–0.99)**
**Endometrial cancer**	History of PE						
	No	2,246,014	6452	1.00 (Ref)	977,523	942	1.00 (Ref)
	Yes	86,269	243	**1.28 (1.13–1.46)**	37,226	55	**1.46 (1.14–1.74)**
**Thyroid cancer**	History of PE						
	No	2,246,014	3609	1.00 (Ref)	977,523	2616	1.00 (Ref)
	Yes	86,269	111	0.85 (0.70-1.02)	37,226	107	1.12 (0.93–1.32)

The incidence rate ratios (IRRs) were estimated using Poisson regression with age as the time scale and adjusting for the woman’s birth year, age at first birth, and parity. PE denotes preeclampsia.

*Cancers include both in situ and invasive cases, except for cervical cancer in Finland, where only invasive cases are recorded in the Cancer Registry.

All bold values are statistically significant results.

Conversely, we found an increased incidence for endometrial cancer in PE women, with an IRR of 1.28 (95% CI: 1.13–1.46) in Sweden and 1.46 (95% CI 1.14–1.74) in Finland. The results were non-significant when assessed in the subgroups for other, more rare cancer types including ovarian cancer, melanoma, thyroid cancer, or colorectal cancer (Table 2).

The sex of the foetus did not significantly influence on the overall risk of cancer development. There was a pronounced effect for endometrial cancer in the Swedish cohort, where PE women carrying a male foetus had an increased incidence with IRR of 1.39 (95% CI: 1.18–1.65). In contrast, the opposite trend was observed in the Finnish cohort, with an IRR of 1.55 (95% CI: 1.18–1.92) in PE women carrying a female foetus (Table 3). In the Finnish cohort, PE women carrying a male foetus had a slightly reduced risk of developing breast cancer, with an IRR of 0.88 (95% CI: 0.77–0.99) (Table 3).

**Table 3:** Association of preeclampsia at the first delivery and offspring sex with subsequent risk of cancer.

		Sweden						Finland					
		With female foetus			With male foetus			With female foetus			With male foetus		
		No. ppl	No. cases	IRR (95% CI)	No. ppl	No. cases	IRR (95% CI)	No. ppl	No. cases	IRR (95% CI)	No. ppl	No. cases	IRR (95% CI)
**Any cancer**	History of PE												
	No	1,091,723	138,890	1.00 (Ref)	1,154,179	146,512	1.00 (Ref)	477,122	24,843	1.00 (Ref)	500,356	25,523	1.00 (Ref)
	Yes	41,346	4289	**0.92 (0.90–0.95)**	44,917	4589	**0.91 (0.88–0.93)**	18,245	855	0.95 (0.88–1.01)	18,981	866	1.00 (0.93–1.07)
**Cervical cancer** ^*^	History of PE												
	No	1,091,723	46,249	1.00 (Ref)	1,154,179	48,434	1.00 (Ref)	477,122	633	1.00 (Ref)	500,356	686	1.00 (Ref)
	Yes	41,346	1367	**0.82 (0.77–0.86)**	44,917	1395	**0.76 (0.72–0.80)**	18,245	16	**0.60 (0.10–1.10)**	18,981	15	**0.50 (0.01–1.01)**
**Breast cancer**	History of PE												
	No	1,091,723	33,540	1.00 (Ref)	1,154,179	35,463	1.00 (Ref)	477,122	9511	1.00 (Ref)	500,356	10,087	1.00 (Ref)
	Yes	41,346	977	**0.90 (0.84–0.95)**	44,917	1062	**0.90 (0.84–0.95)**	18,245	327	0.93 (0.82–1.04)	18,981	324	**0.88 (0.77–0.99)**
**Melanoma**	History of PE												
	No	1,091,723	9721	1.00 (Ref)	1,154,179	10,496	1.00 (Ref)	477,122	1435	1.00 (Ref)	500,356	1478	1.00 (Ref)
	Yes	41,346	358	1.06 (0.96–1.18)	44,917	399	1.07 (0.97–1.18)	18,245	50	0.94 (0.66–1.22)	18,981	49	0.91 (0.63–1.92)
**Colorectal cancer**	History of PE												
	No	1,091,723	7385	1.00 (Ref)	1,154,179	7865	1.00 (Ref)	477,122	479	1.00 (Ref)	500,356	495	1.00 (Ref)
	Yes	41,346	204	0.90 (0.79–1.04)	44,917	247	1.01 (0.89–1.15)	18,245	14	0.78 (0.24–1.31)	18,981	17	0.95 (0.47–1.43)
**Ovarian cancer**	History of PE												
	No	1,091,723	4017	1.00 (Ref)	1,154,179	4258	1.00 (Ref)	477,122	580	1.00 (Ref)	500,356	634	1.00 (Ref)
	Yes	41,346	132	1.04 (0.87–1.24)	44,917	146	1.05 (0.89–1.24)	18,245	18	0.84 (0.37–1.31)	18,981	23	0.99 (0.58–1.41)
**Lung cancer**	History of PE												
	No	1,091,723	4050	1.00 (Ref)	1,154,179	4209	1.00 (Ref)	477,122	570	1.00 (Ref)	500,356	595	1.00 (Ref)
	Yes	41,346	89	**0.78 (0.63–0.96)**	44,917	82	**0.67 (0.54–0.83)**	18,245	14	0.58 (0.05–1.11)	18,981	16	0.68 (0.18–1.18)
**Endometrial cancer**	History of PE												
	No	1,091,723	3145	1.00 (Ref)	1,154,179	3307	1.00 (Ref)	477,122	467	1.00 (Ref)	500,356	474	1.00 (Ref)
	Yes	41,346	105	1.16 (0.95–1.41)	44,917	138	**1.39 (1.18–1.65)**	18,245	30	**1.55 (1.18–1.92)**	18,981	25	1.36 (0.96–1.77)
**Thyroid cancer**	History of PE												
	No	1,091,723	1753	1.00 (Ref)	1,154,179	1856	1.00 (Ref)	477,122	1268	1.00 (Ref)	500,356	1348	1.00 (Ref)
	Yes	41,346	52	0.83 (0.63–1.09)	44,917	59	0.86 (0.67–1.12)	18,245	51	1.11 (0.83–1.39)	18,981	56	1.15 (0.88–1.41)

The incidence rate ratios (IRRs) were estimated using Poisson regression with age as the time scale and adjusting for the woman’s birth year, age at first birth, and parity. PE denotes preeclampsia.

*Cancers include both in situ and invasive cases, except for cervical cancer in Finland, where only invasive cases are recorded in the Cancer Registry.

All bold values are statistically significant results.

When examining the full siblings of Swedish PE women, a slight decrease in the risk of developing cancer later in life was observed, with an IRR of 0.97 (95% CI: 0.95–0.99). This reduction was particularly evident in the lung cancer subgroup, where the incidence rate ratio was 0.86 (95% CI: 0.75–0.98). No significant differences were detected for the incidence of other cancer subtypes (Table 4).

**Table 4:** Association of preeclampsia at the first delivery and risk of cancer in siblings of these women.

		Sweden		
		No. ppl	No. cases	IRR (95% CI)
**Any cancer**	History of PE			
	No	1,950,519	220,735	1.00 (Ref)
	Yes	96,361	9146	**0.97 (0.95–0.99)**
**Cervical cancer**	History of PE			
	No	1,950,519	47,535	1.00 (Ref)
	Yes	96,361	2224	0.96 (0.92–1.00)
**Breast cancer**	History of PE			
	No	1,950,519	22,157	1.00 (Ref)
	Yes	96,361	897	0.95 (0.89–1.02)
**Melanoma**	History of PE			
	No	1,950,519	18,901	1.00 (Ref)
	Yes	96,361	833	1.02 (0.95–1.09)
**Colorectal cancer**	History of PE			
	No	1,950,519	15,979	1.00 (Ref)
	Yes	96,361	619	0.98 (0.90–1.06)
**Ovarian cancer**	History of PE			
	No	1,950,519	2489	1.00 (Ref)
	Yes	96,361	115	1.06 (0.88–1.28)
**Lung cancer**	History of PE			
	No	1,950,519	7016	1.00 (Ref)
	Yes	96,361	230	**0.86 (0.75–0.98)**
**Endometrial cancer**	History of PE			
	No	1,950,519	1910	1.00 (Ref)
	Yes	96,361	78	1.06 (0.84–1.33)
**Thyroid cancer**	History of PE			
	No	1,950,519	2162	1.00 (Ref)
	Yes	96,361	118	1.18 (0.98–1.42)
**Prostate cancer**	History of PE			
	No	1,950,519	32,609	1.00 (Ref)
	Yes	96,361	1159	0.95 (0.90–1.01)

The incidence rate ratios (IRRs) were estimated using Poisson regression with age as the time scale, adjusting for the siblings’ birth year, siblings’ sex and number of full-sibling sisters. PE denotes preeclampsia.

All bold values are statistically significant results.

## Discussion

This population-based study of 3,401,724 women from two countries demonstrates an association between PE at the first delivery and later incidence of cancer. We found a reduced risk of any cancer in the Swedish cohort, while the results did not reach statistical significance in the Finnish cohort. However, when analysed by cancer subtypes, both national cohorts showed similar findings: 9–10% reduction in the risk for breast cancer, 21–45% reduction for cervical cancer, and 28–37% reduction for lung cancer. Notably, an increased risk of 28-46% for endometrial cancer was found in both cohorts, whereas no associations were found for other, more rare cancer subtypes. Although these observational data cannot prove causality, our study proposes that similar mechanisms might contribute to PE and cancer development.

A main strength of our study is the large population-based series from two countries. This allowed us to study 123,495 women with PE and over 3.2 million women without, with the large sample size ensuring adequate statistical power. The national healthcare systems in Sweden and Finland are very similar and publicly funded, and access to healthcare is guaranteed by law. All maternal diagnoses related to the birth are registered, including all complications diagnosed earlier during pregnancy, indications for induction, and indications for caesarean section. In fact, coding of the disorders has been consistent over several decades, meaning that our data are likely to be highly representative of these populations. Since both the Swedish and Finnish Medical Birth and Cancer Registries have full national coverage, and data can be linked at the individual level [22, 23, 27, 28],misclassifications of diagnoses related to delivery or cancer are unlikely. The prevalence of PE in both cohorts (2.8% in Sweden and 2.5% in Finland) is in line with national population estimates, supporting the accuracy of the data. Additionally, we adjusted data on maternal and pregnancy-related characteristics to minimize environmental or time-dependent exposures that might affect cancer risk later in life. Unfortunately, we used a previously built and linked Finnish PE cohort here, and eclampsia cases were missing. On the other hand, the incidence of eclampsia in Finland is very low [29], 1.5 per 10,000 deliveries, meaning that their inclusion would not have major influence in the analyses.

The main limitation of our study is the heterogeneity of PE and challenges in the establishment of explicit diagnostic criteria. PE is characterized by maternal hypertension with abrupt onset after 20 weeks of gestation, accompanied by proteinuria or other related symptoms. Notably, the criteria used for PE diagnosis have changed over the recent decades, and from 2013 onwards [30], proteinuria has not been required for the diagnosis any more. This change, which occurred during the study period, may have influenced coding of PE and its reported incidences. Furthermore, the follow-up period of two decades may still be relatively short to fully evaluate cancer risk. This is suggested by the low number of cancers detected after PE, especially considering that most cancers develop at an advanced age. Migration data, available only for Sweden, limited our ability to track all potential cancer cases in the Finnish cohort. Moreover, we cannot rule out effects of confounders such as body mass index (BMI), smoking, postmenopausal status, hormonal therapy, and lifestyle on subsequent cancer risk. Although, other studies that adjusted for BMI [31] and smoking [13] had unchanged estimates concerning the development of breast cancer and all cancers, respectively. Since the overall cancer incidences in Finland and Sweden are similar, the observed increased risk of getting cancer in the Swedish cohort might be explained by the advanced age and slightly longer follow-up period.

During the past decade, only one other cohort study has assessed the overall risk of cancer following PE. Serrand et al. reported a hazard ratio of 0.94 (95% CI 0.84–1.05) for cancer development following PE; this result did not reach statistical significance likely due to the short mean follow-up period of 4.4 years [32]. A systematic review encompassing 13 studies from 1947 to 2013 found no change in the overall cancer risk among women with PE (RR: 0.98, 95% CI 0.85–1.12) [33]. However, after performing a sensitivity analysis to adjust for heterogeneity, the relative cancer risk was significantly decreased (RR: 0.90, 95% CI 0.85–0.96), in agreement with the findings of our study.

Several studies have demonstrated an inverse association between PE and later risk of breast cancer [7–9, 19, 20], consistent with our result findings. The underlying causes of the potential association between PE and breast cancer are often proposed to be hormonal in nature. Potentially, the lower levels of oestrogens and higher levels of androgens and progesterone in PE might be protective against the development of breast cancer [9, 34].

Research on cervical cancer with respect to PE are both rare and inconsistent [13, 32, 35]. It is well known that, unlike most other cancers, cervical cancer has a single cause, developing as a result of immune evasion by the human papillomavirus (HPV) [36]. In this context, our findings of lower risk of cervical cancer after PE suggest that immune regulation in these women, such as elevated TNF-α, IL-6, and IL-7 responses along with enhanced cytotoxic T-cell activation [37–43], might reduce the risk of persistent HPV infection.

In addition to cervical cancer, lung cancer emerged as a notable cancer subtype in our findings. Unlike cervical cancer, lung cancer is multifactorial, driven by germline genetic variation, environmental exposures like cigarette smoking, and the accumulation of somatic genetic mutations [44]. Both women with PE and their siblings showed a decreased risk of developing lung cancer, with siblings exhibiting a 14% reduced risk. These findings suggest that genetic susceptibility involved in the development of PE may also play a protective role against lung cancer.

Contrary to our hypothesis, our data revealed that PE women had a 28–46% increased risk of developing endometrial cancer. This finding agrees with a case-control study by Trabert et al., reporting higher odds of 43% for endometrial cancer in women with PE [45]. However, other factors, such as having four or more pregnancies, were associated with a reduced risk of endometrial cancer. While some studies found no significant association [11, 46, 47], they observed other relevant variables increasing the odds for endometrial cancer, namely early onset PE [11] and gestational diabetes or newborns large for gestational age [47].

When analysing the impact of fetal sex on cancer development, we observed a pronounced effect in one cancer group. PE women carrying a male foetus had an increased risk of developing endometrial cancer in the Swedish cohort and vice versa in the Finnish cohort. During the embryonic stage, male fetuses are exposed to higher levels of androgens compared to female fetuses; furthermore, the male foetus produces androgens of its own from the testes [48]. It is proposed that androgens are associated with the development of endometrial carcinogenesis, either by transformation to oestrogens or by inhibiting the protective effects of progesterone [49, 50]. This phenomena was observed in a study on twins where mothers with twin boys, compared to twin girls and sex non-concordant twins, had a higher risk of developing endometrial cancer primarily after the age of 55 [51]. The opposing trend observed in the Finnish cohort, combined with the study design and the rare prevalence of the cancer form, limits our ability to draw any conclusions about the influence of fetal sex on the development of endometrial cancer. Although, this finding partially supports the notion that hormones may not be the sole common factor linking PE and cancer as previously proposed [34].

Our theory that an inherited response may link PE and cancer is supported by a prior study showing: 1. a reduced risk of PE in sisters of breast cancer patients and 2. lower mammographic breast density in sisters of PE women [8]. To the best of our knowledge, this study is the first to analyse the overall cancer risk in siblings of women with PE. Indeed, in the Swedish cohort, the overall cancer incidence was reduced in siblings of PE women, however the effect was very modest.

In conclusion, our study implicates that women with PE in their first pregnancy possess lower risk of breast, cervical, and lung cancer later in life. In contrast, the risk of endometrial cancer is increased after PE. These data suggest that maternal characteristics or pregnancy-induced changes, may alter the chances of developing cancer later in life.

## Data Availability

The data that support the findings of this study are not openly available due to reasons of sensitivity and are available from the corresponding author upon reasonable request. Data are located in controlled access data storage at Karolinska Institutet.

## References

[1] Gestational Hypertension and Preeclampsia. ACOG practice bulletin summary, number 222. Obstet Gynecol. 2020;135:1492–5. 32443077 10.1097/AOG.0000000000003892

[2] Garovic VD, White WM, Vaughan L, Saiki M, Parashuram S, Garcia-Valencia O, et al. Incidence and long-term outcomes of hypertensive disorders of pregnancy. J Am Coll Cardiol. 2020;75:2323–34. 32381164 10.1016/j.jacc.2020.03.028 PMC7213062

[3] Brown DW, Dueker N, Jamieson DJ, Cole JW, Wozniak MA, Stern BJ, et al. Preeclampsia and the risk of ischemic stroke among young women: results from the Stroke Prevention in Young Women Study. Stroke. 2006;37:1055–9. 16484606 10.1161/01.STR.0000206284.96739.ee

[4] Vikse BE, Hallan S, Bostad L, Leivestad T, Iversen BM. Previous preeclampsia and risk for progression of biopsy-verified kidney disease to end-stage renal disease. Nephrol Dial Transpl. 2010;25:3289–96. 10.1093/ndt/gfq169 20348149

[5] Davis EF, Lewandowski AJ, Aye C, Williamson W, Boardman H, Huang RC, et al. Clinical cardiovascular risk during young adulthood in offspring of hypertensive pregnancies: insights from a 20-year prospective follow-up birth cohort. BMJ Open. 2015;5:e008136. 26105032 10.1136/bmjopen-2015-008136 PMC4480003

[6] Kajantie E, Eriksson JG, Osmond C, Thornburg K, Barker DJ. Pre-eclampsia is associated with increased risk of stroke in the adult offspring: the Helsinki birth cohort study. Stroke. 2009;40:1176–80. 19265049 10.1161/STROKEAHA.108.538025

[7] Pacheco NL, Andersen AM, Kamper-Jørgensen M. Preeclampsia and breast cancer: the influence of birth characteristics. Breast. 2015;24:613–7. 26144638 10.1016/j.breast.2015.06.006

[8] Yang H, He W, Eriksson M, Li J, Holowko N, Chiesa F, et al. Inherited factors contribute to an inverse association between preeclampsia and breast cancer. Breast Cancer Res. 2018;20:6. 29361985 10.1186/s13058-017-0930-6 PMC5782395

[9] Wright LB, Schoemaker MJ, Jones ME, Ashworth A, Swerdlow AJ. Breast cancer risk in relation to history of preeclampsia and hyperemesis gravidarum: Prospective analysis in the Generations Study. Int J Cancer. 2018;143:782–92. 29516507 10.1002/ijc.31364 PMC6055869

[10] Kim JS, Kang EJ, Woo OH, Park KH, Woo SU, Yang DS, et al. The relationship between preeclampsia, pregnancy-induced hypertension and maternal risk of breast cancer: a meta-analysis. Acta Oncol. 2013;52:1643–8. 23240638 10.3109/0284186X.2012.750033

[11] Hallum S, Pinborg A, Kamper-Jørgensen M. Long-term impact of preeclampsia on maternal endometrial cancer risk. Br J Cancer. 2016;114:809–12. 26964032 10.1038/bjc.2016.55 PMC4984869

[12] Abril J, Trabert B, Troisi R, Grotmol T, Ekbom A, Engeland A, et al. Associations between pregnancy-related factors and birth characteristics with risk of rare uterine cancer subtypes: a Nordic population-based case-control study. Cancer Causes Control. 2024;35:741–7. 10.1007/s10552-023-01832-6 PMC11441445 38129544

[13] Calderon-Margalit R, Friedlander Y, Yanetz R, Deutsch L, Perrin MC, Kleinhaus K, et al. Preeclampsia and subsequent risk of cancer: update from the Jerusalem Perinatal Study. Am J Obstet Gynecol. 2009;200:63.e1–5. 18822400 10.1016/j.ajog.2008.06.057 PMC2660849

[14] Boulanger H, Bounan S, Mahdhi A, Drouin D, Ahriz-Saksi S, Guimiot F, et al. Immunologic aspects of preeclampsia. AJOG Glob Rep. 2024;4:100321. 38586611 10.1016/j.xagr.2024.100321 PMC10994979

[15] Wedenoja S, Yoshihara M, Teder H, Sariola H, Gissler M, Katayama S, et al. Fetal HLA-G mediated immune tolerance and interferon response in preeclampsia. EBioMedicine. 2020;59:102872. 32680723 10.1016/j.ebiom.2020.102872 PMC7502669

[16] Hiam-Galvez KJ, Allen BM, Spitzer MH. Systemic immunity in cancer. Nat Rev Cancer. 2021;21:345–59. 33837297 10.1038/s41568-021-00347-z PMC8034277

[17] Kareva I. Immune suppression in pregnancy and cancer: parallels and insights. Transl Oncol. 2020;13:100759. 32353791 10.1016/j.tranon.2020.100759 PMC7191218

[18] Deer E, Herrock O, Campbell N, Cornelius D, Fitzgerald S, Amaral LM, et al. The role of immune cells and mediators in preeclampsia. Nat Rev Nephrol. 2023;19:257–70. 36635411 10.1038/s41581-022-00670-0 PMC10038936

[19] Opdahl S, Romundstad PR, Alsaker MDK, Vatten LJ. Hypertensive diseases in pregnancy and breast cancer risk. Br J Cancer. 2012;107:176–82. 22576589 10.1038/bjc.2012.195 PMC3389406

[20] Vatten LJ, Forman MR, Nilsen TI, Barrett JC, Romundstad PR. The negative association between pre-eclampsia and breast cancer risk may depend on the offspring’s gender. Br J Cancer. 2007;96:1436–8. 17387346 10.1038/sj.bjc.6603688 PMC2360175

[21] Sun M, Fan Y, Hou Y, Fan Y. Preeclampsia and maternal risk of breast cancer: a meta-analysis of cohort studies. J Matern Fetal Neonatal Med. 2018;31:2484–91. 28715959 10.1080/14767058.2017.1342806

[22] Cnattingius S, Källén K, Sandström A, Rydberg H, Månsson H, Stephansson O, et al. The Swedish medical birth register during five decades: documentation of the content and quality of the register. Eur J Epidemiol. 2023;38:109–20. 36595114 10.1007/s10654-022-00947-5 PMC9867659

[23] Gissler M, Teperi J, Hemminki E, Meriläinen J. Data quality after restructuring a national medical registry. Scand J Soc Med. 1995;23:75–80. 7784857 10.1177/140349489502300113

[24] Albrektsen G, Heuch I, Hansen S, Kvåle G. Breast cancer risk by age at birth, time since birth and time intervals between births: exploring interaction effects. Br J Cancer. 2005;92:167–75. 15597097 10.1038/sj.bjc.6602302 PMC2361726

[25] Adami HO, Hsieh CC, Lambe M, Trichopoulos D, Leon D, Persson I, et al. Parity, age at first childbirth, and risk of ovarian cancer. Lancet. 1994;344:1250–4. 7967985 10.1016/s0140-6736(94)90749-8

[26] Nichols HB, Schoemaker MJ, Cai J, Xu J, Wright LB, Brook MN, et al. Breast cancer risk after recent childbirth: a pooled analysis of 15 prospective studies. Ann Intern Med. 2019;170:22–30. 30534999 10.7326/M18-1323 PMC6760671

[27] Barlow L, Westergren K, Holmberg L, Talbäck M. The completeness of the Swedish Cancer Register: a sample survey for year 1998. Acta Oncol. 2009;48:27–33. 18767000 10.1080/02841860802247664

[28] Leinonen MK, Miettinen J, Heikkinen S, Pitkäniemi J, Malila N. Quality measures of the population-based Finnish Cancer Registry indicate sound data quality for solid malignant tumours. Eur J Cancer. 2017;77:31–9. 28350996 10.1016/j.ejca.2017.02.017

[29] Jaatinen N, Ekholm E. Eclampsia in Finland; 2006 to 2010. Acta Obstetricia et Gynecologica Scand. 2016;95:787–92. 10.1111/aogs.12882 26919049

[30] Hypertension in pregnancy. Report of the American College of Obstetricians and Gynecologists’ task force on hypertension in pregnancy. Obstet Gynecol. 2013;122:1122–31. 24150027 10.1097/01.AOG.0000437382.03963.88

[31] Ma H, Henderson KD, Sullivan-Halley J, Duan L, Marshall SF, Ursin G, et al. Pregnancy-related factors and the risk of breast carcinoma in situ and invasive breast cancer among postmenopausal women in the California Teachers Study cohort. Breast Cancer Res. 2010;12:R35. 20565829 10.1186/bcr2589 PMC2917030

[32] Serrand C, Mura T, Fabbro-Peray P, Seni G, Mousty È, Boudemaghe T, et al. Assessment of All-Cause Cancer Incidence Among Individuals With Preeclampsia or Eclampsia During First Pregnancy. JAMA Netw Open. 2021;4:e2114486. 34160606 10.1001/jamanetworkopen.2021.14486 PMC8223101

[33] Wang F, Zhang W, Cheng W, Huo N, Zhang S. Preeclampsia and cancer risk in women in later life: a systematic review and meta-analysis of cohort studies. Menopause. 2021;28:1070–8. 34374685 10.1097/GME.0000000000001806

[34] Nechuta S, Paneth N, Velie EM. Pregnancy characteristics and maternal breast cancer risk: a review of the epidemiologic literature. Cancer Causes Control. 2010;21:967–89. 20224871 10.1007/s10552-010-9524-7 PMC3863387

[35] Walfisch A, Kessous R, Davidson E, Sergienko R, Sheiner E. Pre-eclampsia and future female malignancy. Hypertens Pregnancy. 2015;34:456–63. 26390054 10.3109/10641955.2015.1071838

[36] Perkins RB, Wentzensen N, Guido RS, Schiffman M. Cervical cancer screening: a review. Jama. 2023;330:547–58. 37552298 10.1001/jama.2023.13174

[37] Prins JR, Faas MM, Melgert BN, Huitema S, Timmer A, Hylkema MN, et al. Altered expression of immune-associated genes in first-trimester human decidua of pregnancies later complicated with hypertension or foetal growth restriction. Placenta. 2012;33:453–5. 22386644 10.1016/j.placenta.2012.02.010

[38] Aggarwal R, Jain AK, Mittal P, Kohli M, Jawanjal P, Rath G. Association of pro- and anti-inflammatory cytokines in preeclampsia. J Clin Lab Anal. 2019;33:e22834. 30666720 10.1002/jcla.22834 PMC6528584

[39] Szarka A, Rigó J Jr., Lázár L, Beko G, Molvarec A. Circulating cytokines, chemokines and adhesion molecules in normal pregnancy and preeclampsia determined by multiplex suspension array. BMC Immunol. 2010;11:59. 21126355 10.1186/1471-2172-11-59 PMC3014878

[40] Gadonski G, LaMarca BB, Sullivan E, Bennett W, Chandler D, Granger JP. Hypertension produced by reductions in uterine perfusion in the pregnant rat: role of interleukin 6. Hypertension. 2006;48:711–6. 16940225 10.1161/01.HYP.0000238442.33463.94

[41] Formby B. Immunologic response in pregnancy. Its role in endocrine disorders of pregnancy and influence on the course of maternal autoimmune diseases. Endocrinol Metab Clin North Am. 1995;24:187–205. 7781626

[42] Madazli R, Aydin S, Uludag S, Vildan O, Tolun N. Maternal plasma levels of cytokines in normal and preeclamptic pregnancies and their relationship with diastolic blood pressure and fibronectin levels. Acta Obstet Gynecol Scand. 2003;82:797–802. 12911439 10.1034/j.1600-0412.2003.00206.x

[43] LaMarca BB, Cockrell K, Sullivan E, Bennett W, Granger JP. Role of endothelin in mediating tumor necrosis factor-induced hypertension in pregnant rats. Hypertension. 2005;46:82–6. 15928030 10.1161/01.HYP.0000169152.59854.36

[44] Sampson JN, Wheeler WA, Yeager M, Panagiotou O, Wang Z, Berndt SI, et al. Analysis of Heritability and Shared Heritability Based on Genome-Wide Association Studies for Thirteen Cancer Types. J Natl Cancer Inst. 2015;107:djv279. 26464424 10.1093/jnci/djv279 PMC4806328

[45] Trabert B, Troisi R, Grotmol T, Ekbom A, Engeland A, Gissler M, et al. Associations of pregnancy-related factors and birth characteristics with risk of endometrial cancer: A Nordic population-based case-control study. Int J Cancer. 2020;146:1523–31. 31173648 10.1002/ijc.32494 PMC6898733

[46] Jordao H, Herink K, Ka E, McVicker L, Kearns C, McMenamin ÚC. Pre-eclampsia during pregnancy and risk of endometrial cancer: a systematic review and meta-analysis. BMC Women’s Health. 2023;23:259. 37173714 10.1186/s12905-023-02408-x PMC10182685

[47] Liu Y, Chen X, Sheng J, Sun X, Chen GQ, Zhao M, et al. Complications of pregnancy and the risk of developing endometrial or ovarian cancer: a case-control study. Front Endocrinol (Lausanne). 2021;12:642928. 33995276 10.3389/fendo.2021.642928 PMC8121171

[48] Auyeung B, Baron-Cohen S, Ashwin E, Knickmeyer R, Taylor K, Hackett G, et al. Fetal testosterone predicts sexually differentiated childhood behavior in girls and in boys. Psychol Sci. 2009;20:144–8. 19175758 10.1111/j.1467-9280.2009.02279.x PMC2778233

[49] McGrath M, Lee IM, Hankinson SE, Kraft P, Hunter DJ, Buring J, et al. Androgen receptor polymorphisms and endometrial cancer risk. Int J Cancer. 2006;118:1261–8. 16161040 10.1002/ijc.21436

[50] Kaaks R, Lukanova A, Kurzer MS. Obesity, endogenous hormones, and endometrial cancer risk: a synthetic review. Cancer Epidemiol Biomark Prev. 2002;11:1531–43. 12496040

[51] Albrektsen G, Heuch I, Thoresen S, Kvåle G. Twin births, sex of children and maternal risk of endometrial cancer: a cohort study in Norway. Acta Obstet Gynecol Scand. 2008;87:1123–8. 18951203 10.1080/00016340802443780

